# Spatial and temporal variation in the community prevalence of antibiotic resistance in Bangladesh: an integrated surveillance study protocol

**DOI:** 10.1136/bmjopen-2018-023158

**Published:** 2018-04-28

**Authors:** Emily Rousham, Leanne Unicomb, Paul Wood, Michael Smith, Muhammad Asaduzzaman, Mohammad Aminul Islam

**Affiliations:** 1 Centre for Global Health and Human Development, School of Sport, Exercise and Health Sciences, Loughborough University, Loughborough, UK; 2 Environmental Intervention Unit, Infectious Diseases Division, International Centre for Diarrhoeal Disease Research, Bangladesh, Dhaka, Bangladesh; 3 Department of Geography, School of Social, Political and Geographical Sciences, Loughborough University, Loughborough, UK; 4 Water, Engineering and Development Centre, School of Architecture, Building and Civil Engineering, Loughborough University, Loughborough, UK; 5 Laboratory Sciences and Services Division, International Centre for Diarrhoeal Disease Research, Bangladesh, Dhaka, Bangladesh

**Keywords:** public health, epidemiology, diagnostic microbiology

## Abstract

**Introduction:**

Increasing antibiotic resistance (ABR) in low-income and middle-income countries such as Bangladesh presents a major health threat. However, assessing the scale of the health risk is problematic in the absence of reliable data on the community prevalence of antibiotic-resistant bacteria. We describe the protocol for a small-scale integrated surveillance programme that aims to quantify the prevalence of colonisation with antibiotic-resistant bacteria and concentrations of antibiotic-resistant genes from a ‘One Health’ perspective. The holistic assessment of ABR in humans, animals and within the environment in urban and rural Bangladesh will generate comprehensive data to inform human health risk.

**Methods and analysis:**

The study design focuses on three exposure-relevant sites where there is enhanced potential for transmission of ABR between humans, animals and the environment: (1) rural poultry-owning households, (2) commercial poultry farms and (3) urban live-bird markets. The comparison of ABR prevalence in human groups with high and low exposure to farming and poultry will enable us to test the hypothesis that ABR bacteria and genes from the environment and food-producing animals are potential sources of transmission to humans. *Escherichia coli* is used as an ABR indicator organism due to its widespread environmental presence and colonisation in both the human and animal gastrointestinal tract.

**Ethics and dissemination:**

The study has been approved by the Institutional Review Board of the International Centre for Diarrhoeal Disease Research, Bangladesh, and Loughborough University Ethics Committee. Data for the project will be stored on the open access repository of the Centre for Ecology and Hydrology, Natural Environment Research Council. The results of this study will be published in peer-reviewed journals and presented at national and international conferences.

**Strengths and limitations of this study**

- We present a study protocol focused on integrated surveillance of antibiotic resistance in urban and rural Bangladesh using a One Health approach.
- Assessment of the human, poultry and environmental prevalence of antibiotic-resistant *Escherichia coli* will identify potential hot spots for its transmission in Bangladesh.
- Seasonal and spatial variation on the occurrence of antibiotic-resistant bacteria in humans, poultry and the wider environment will be assessed.
- The two regions included in the study may not be typical of all regions within Bangladesh.

## Introduction

Antibiotic resistance (ABR) has been widely recognised as an emerging threat to human health and a global research priority for capacity development and public health campaigns in clinical and community settings.^1^ Antimicrobial resistance is seen as a ‘One Health’ challenge.^1^ That is, efforts are needed to tackle the problem of ABR in all three domains of humans, animals and in the wider environment in order to achieve optimal human health.^2^

The natural environment is acknowledged as an important reservoir of antibiotic-resistant bacteria, with anthropogenic activities such as agriculture, antibiotic residues and wastewater disposal potentially enhancing the horizontal transfer of resistant genes.^3 4^ Genes for ABR can survive in the environment, collectively known as the ‘resistome’, and the exchange of genetic material encoding for antimicrobial resistance can take place through mobile genetic elements such as plasmids via conjugation and other mechanisms in environmental compartments.^5^ Antimicrobials have been shown also to induce the transfer of resistance determinants between micro-organisms.^6^ These processes may be accelerated by the presence of environmental contaminants or pollutants such as heavy metals, antibiotic residues or biocides.^7^ However, it is not yet known to what extent these environmental sources of ABR may be transmitted to humans. There is therefore a pressing need for research to quantify the extent of transmission of clinically relevant ABR bacteria from the environment to humans.^8^

Resistance to third-generation cephalosporins is increasing as bacteria acquire the ability to produce extended-spectrum beta-lactamases (ESBLs). The prevalence of human colonisation with ESBL-producing *Escherichia coli* is estimated to be around 14% globally, with rates as high as 22% in Asia and Sub-Saharan Africa.^9^ Of greater concern, however, is the emergence and spread of carbapenem resistance among clinically relevant bacteria via genes such as New Delhi Metallo-β-lactamase-1 (NDM-1) and colistin resistance via *mcr*-1 gene.^10–12^ The detection of these genes in humans, animals and the environment has led to heightened concern regarding the environment as a major transmission route for antimicrobial resistance to humans.^10^ NDM-1 has been identified in Bangladesh since 2010,^13^ after the first detection and rapid spread across India and Pakistan from 2008 onwards.^14 15^ Municipal water supplies in Dhaka are known to carry high levels of sewage-derived bacteria.^16^ Around 36% of *E. coli* isolates from tap water samples in Dhaka city were reported to be multidrug-resistant and 26% of these were positive for ESBLs, posing a serious threat to public health.^16^ Drinking water contamination is also common in rural areas of Bangladesh; 59% of drinking water samples for children that were stored or directly obtained mostly from tubewells were positive for *E. coli* faecal contamination^17^ but were not tested for resistant phenotypes. Prospective sampling indicated that ingestion of contaminated water led to subsequent episodes of diarrhoea among children.^17^ Approximately 3.5% of Gram-negative clinical isolates in Bangladesh are NDM-1-producing,^18^ and faecal carriage of NDM-1-producing bacteria has been demonstrated among patients with diarrhoea.^13 19^

The physical and social environment in Bangladesh favours the rapid spread of ABR with high-density populations, lack of clean drinking water and poor sanitation infrastructure. Widespread faecal contamination in water and soil, as well as human hands and food, has been demonstrated in microbiological studies in Bangladesh.^20^ The availability of inexpensive antimicrobials from over-the-counter suppliers leads to inappropriate use of antibiotics.^21^ The release of antimicrobials into the environment through drug manufacturing and poor waste disposal processes adds further complexities to the pathways for the selection and spread of resistance in the environment.^22^ In Bangladesh, a high prevalence of *bla*_NDM-1_ genes has been found in hospital wastewater compared with community wastewater in the city of Dhaka, highlighting anthropogenic influences on ABR prevalence.^23^

As well as the risk factors for ABR associated with water supply, sanitation and infrastructure, the environmental risks of ABR transmission are likely to change with seasonal and rainfall variability, and concomitant changes in human exposure risk to contaminated surface and drinking water.^20^ Seasonal behaviours may also change transmission dynamics. Ahammad *et al*^24^ found high concentrations of *bla*_NDM-1_ in the Yamuna River in Delhi and increased concentration of *bla*_NDM-1_ in rural river water and sediment samples following the mass pilgrimage of visitors from New Delhi to a holy site in Rishikesh-Haridwar, North India.

We have identified the following gaps in knowledge surrounding the prevalence and transmission of ABR genes and bacteria at the community level: (1) an absence of contemporaneous sampling of ABR in humans, animals and the wider environment in low-income and middle-income countries such as Bangladesh; (2) a lack of quantitative measures of ABR bacteria (*E. coli*) and resistance genes in potential hot spots for transmission at an appropriate spatial and temporal scale; (3) an absence of baseline data from rural locations to establish whether ABR prevalence rates are similar to those observed in urban Dhaka; (4) a lack of knowledge regarding the seasonal dynamics of resistant bacteria and gene concentrations in different environmental compartments; and (5) a lack of comparative data of human carriage of resistant genes in groups with high and low exposure to contaminated outdoor environments.

The specific objectives of the research are as follows:

1. To ascertain the prevalence and concentration of antibiotic-resistant bacteria (*E. coli*) and resistance genes for ESBLs and carbapenemases (*bla*_CTX-M-1_ and *bla*_NDM-1_) among humans, animal reservoirs (poultry) and different environmental compartments (water, soil and waste) in Bangladesh.
2. To identify the selective drivers of ABR in three exposure-relevant outdoor environments: urban poultry markets, commercial poultry farms and rural villages.
3. To assess the transfer of resistance within and between microbial communities by comparing ABR carriage rates in humans with high and low exposure to environmental and animal reservoirs of ABR genes.
4. To assess the anthropogenic influences on ABR in the natural environment through ethnographic research among poultry farmers, market sellers and village poultry owners.

To explore the transmission of ABR between the outdoor environment and human and animal hosts, we consider poultry as one of the key drivers of ABR selection through faecal shedding of ABR bacteria. Poultry have high rates of faecal carriage of ABR bacteria; they are the most common domestic animal in rural areas of Bangladesh,^25^ as well as being reared in urban slums in Bangladesh.^26^ Among poultry owners in rural Bangladesh, 57% reported using over-the-counter antibiotics for their poultry in the last 6 months, 50% reported sharing their sleeping area with poultry and 61% used poultry faeces as fertiliser.^25^ The rapid expansion of commercial poultry farming in periurban areas carries risks for ABR, with high rates of antibiotic use for growth promotion and prophylactic treatment and poor regulation of waste disposal.^27 28^ Fresh food and poultry markets in Dhaka city are the largest outlets of commercial poultry where birds are slaughtered and processed on site with no regulated solid and liquid waste disposal system.^29^ Faecal wastes produced within commercial farms and urban markets are mostly disposed into the environment through direct washout.^29^

## Methods and analysis

**Figure 1:**
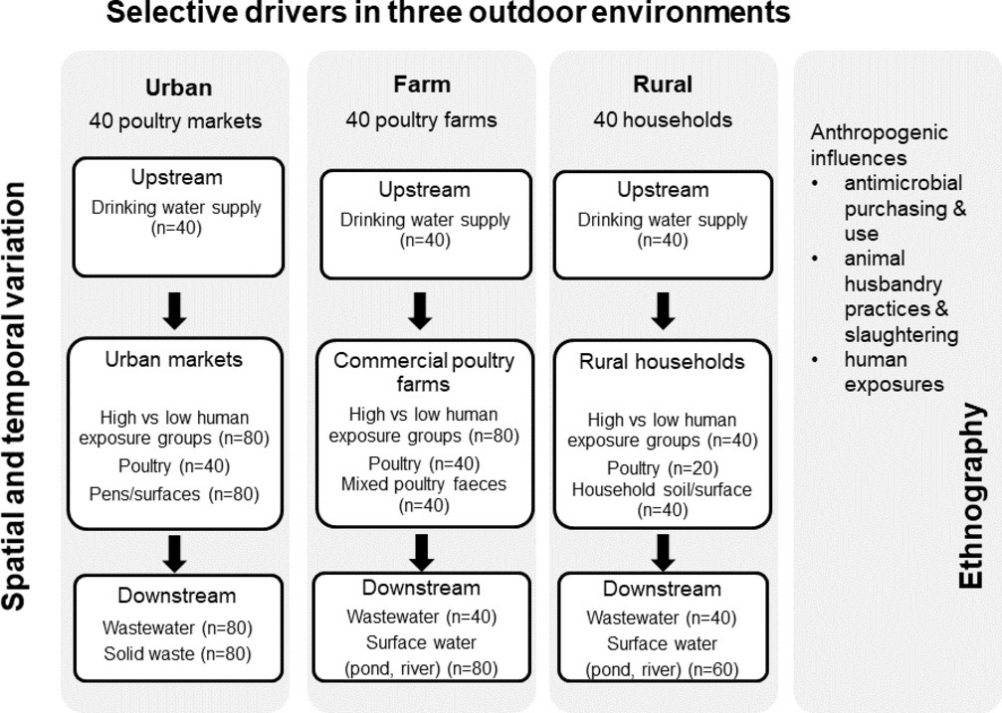
Sampling framework showing the three study sites in relation to the research objectives. In each site, one sampling round will be conducted in the dry season (n=20 markets; n=20 farms; n=20 households) and another sampling round in the wet season (an additional n=20 markets; n=20 farms; n=20 households).

The conceptual framework for the study design and the sampling strategy is shown in figure 1. The study will generate robust data through a sampling framework targeting three distinct outdoor environments with a high potential for ABR transmission: (1) urban live poultry markets in high-density residential areas, (2) commercial poultry farms in rural sites serving the urban retail market, and (3) rural villages with traditional animal husbandry and backyard poultry.

*E. coli* will be investigated as an indicator organism due to its reliability, ease of culturing, affordability and relevance as a contributor to both diarrhoeal disease (eg, diarrhoeagenic strains) and ABR (eg, multidrug-resistant strains). *E. coli* has been listed by the WHO as a critically important organism for antimicrobial resistance.^30^ The bacterium is a normal resident of the intestinal tract of both human and animals, as well as the environment, but pathogenic strains give rise to clinical diseases in both hosts.^31^ Hence, *E. coli* is widely used in studies of the environmental transmission of ABR, particularly for community-acquired resistance using a One Health approach.^4^

The geospatial nature of the transfer of resistance genes will be assessed through geospatial mapping of concentrations of resistant genes and bacteria in upstream localities (water supply), sites close to contamination (source) and downstream (surface waters, soil and solid waste) (see figure 1) at each location using geographical information system software (ArcGIS).

Contemporaneous measures will be taken of healthy humans and animal faecal samples. Temporal variability will be assessed by comparison of resistant *E. coli* bacteria and gene concentrations (*bla*_NDM-1_ and *bla*_CTX-M-1_) between the dry and wet seasons in environmental compartments as well as in the human and animal species.

Ethnographic data will be collected regarding customs and practices of market poultry sellers, slaughterers, farm workers and village households that own domestic chickens. Initially, formative observations will be carried out by a trained field team led by an anthropologist, followed by indepth interviews with individuals who work in poultry markets, commercial farms (farmers and suppliers of chicks and feed) or who own backyard/domestic poultry (12–15 interviews of 30–40 min duration). The formative observations and indepth interviews will identify key behaviours relating to human–poultry interactions in each environmental location. Following the formative observations and indepth interviews, structured observations will be performed to measure behaviours leading to human exposure to poultry and environmental sources of potential contamination. This will yield quantitative data relating to poultry handling, hand washing before and after handling poultry, poultry meat and fresh food preparation, water use, and the disposal of solid waste material (poultry faeces and carcasses/feathers). This information, alongside qualitative data from indepth interviews, will provide contextual data on the human behaviours and practices that may increase environmental contamination and/or increase human exposure to contaminated environments and animals.

An interviewer-administered survey of participants will document common practices of domestic (rural) and commercial poultry holders (farmers and market sellers). Domestic and commercial poultry owners and market sellers will be questioned about antimicrobial additives in feed, purchase of antimicrobial treatment for animals, and the personal purchase and use of antibiotics for themselves.

### Selection and recruitment of study participants

Human participants will be recruited to take part in the study on a voluntary basis and will be provided with information about the study. Only those who volunteer and provide their written informed consent will take part in surveys and provide a faecal sample. The sampling within live poultry markets will start from the centre of a market, omitting the first 10 eligible stalls, approaching the next eligible stall and asking the owner to participate. In this way, stallholders with high exposure and their poultry will be recruited. The low exposure group will comprise stallholders without animal produce (eg, those selling grocery items/fruits/vegetables) in the same markets. The same geographical sampling strategy will be used for household selection in villages to attain 10 poultry-owning and 10 non-poultry-owning households. Farms will be selected from a list registered with the Department of Livestock Services and access requested through the Upazila Livestock Officer. A low-risk comparison group of non-farm workers will be recruited from the same residential area as the farm workers. A team of four fieldworkers will collect samples and data in the wet (monsoon) and dry seasons. Supervision and coordination of fieldwork and laboratory analysis of samples will be carried out by an experienced research investigator. Data collection commenced in March 2017 and is ongoing.

### Patient and public involvement

As this was a study of healthy adults in community settings, a patient group was not involved in the study design or research protocol. However, the study team consulted local community representatives prior to commencing fieldwork to discuss the research, consulted with the main local hospital, and liaised with local government staff in livestock services to discuss working with the poultry farm workers, as well as obtain a list of registered commercial farms. This did not result in any direct changes to the protocol but confirmed that the intended methods and approaches were appropriate. Feedback on the research findings will be provided through open meetings in the communities, as well as sharing with relevant stakeholders and users of the research as part of the planned dissemination activities.

### Sample collection for laboratory analysis

#### Poultry caecal samples

Live chickens will be collected from rural households, poultry farms and live-bird markets, and slaughtered. After defeathering, the whole carcass will be placed in a sterile zipper bag. After transportation to the laboratory under a cold chain (4°C–8°C), caecal samples will be collected aseptically from the dissected whole carcass.

#### Poultry faeces samples

Poultry litter samples will be collected from three different areas of the bird cage/container, floor (in cases where birds are kept in a confined area and not in a cage) or grounds of a compound for free-ranging animals, and pooled in a sterile plastic bag and mixed thoroughly.

#### Solid waste samples

Solid waste samples will be collected from the municipal waste disposal sites near the markets. Samples will be collected from three different locations surrounding the waste collection area. A sterile spatula or spoon will be used to collect solid waste (dark soil) to a depth of 2.5–5.0 cm, pooled in a sterile plastic bag and mixed thoroughly.

#### Soil samples

Soil samples will be collected from within rural compounds/households. For households with internal floors made of mud-compacted soil, a sample will be collected from the main room of the house where most people usually sleep. The area should be dry and free of visible faeces, food or waste. In houses with concrete floors, we will choose an area in the yard where there is frequent movement of people (eg, near the kitchen or in front of the main household door). Using an autoclaved spoon, a maximum depth of about 1.5 cm below the soil surface will be excavated covering an area of ~30 cm^2^. Around 150 g of soil will be collected in a labelled sterile sample bag. Using the end of the spoon, a small scoop (about 0.25 g) of soil from the same area will be placed into a cryovial with LifeGuard solution.

#### Wastewater samples

Wastewater samples will be collected from three separate locations of the run-off drain adjacent to the farm, household or market, and pooled together in a sterile plastic bottle (Nalgene, Rochester, New York). Approximately 300 mL of water will be collected from each point by submerging a sterile container into the drain and transferred to a 1000 mL sterile plastic bottle.

#### Surface water samples

Water samples from rivers, ponds or other surface water downstream of periurban and rural areas will be collected using a sterile plastic bottle. A bottle will be filled by plunging downwards about 30 cm below the water surface and resealed tightly to prevent leakage. Particular care will be taken to avoid bacterial contamination inside the bottle or cap from sources other than the water. Sample bottles will be transported to the laboratory within 8 hours of collection.

#### Supply water samples

To collect water sample from tubewells, the hand pump will be operated continuously for 5 min. The mouth of the pump will then be heated by means of a gas torch, followed by pumping several litres of water to waste. A sample of water will be collected aseptically by allowing the water from the pump to flow directly into a sterile bottle. After collection, the cap of the bottle will be replaced carefully.

To collect samples from taps, the outside nozzle of the tap will be cleaned carefully. The tap will then be turned on full and the water allowed to run to waste for 1 min. The outside of the tap will be sterilised using the flame of a gas torch and allowed to cool by running the water to waste for a few seconds. The sterile sample bottle will be filled from a gentle flow of water and the cap placed on the bottle. Sample bottles will be transported to the laboratory within 8 hours of collection.

#### Human stool samples

Each study participant will be provided with a stool sample container and the procedure for the collection of samples will be explained. Field staff will pick up the sample container within 2 hours of collection.

For all solid and liquid samples collected in the field, sample bags, sample bottles and phials will be immediately placed in a cold box (4°C–8°C) and transported to the laboratory for analysis.

### Laboratory processing and analysis of human, animal and environmental samples

DNA will be extracted directly from samples using the following kits: (1) MO BIO PowerWater DNA Isolation Kit (MO BIO Laboratories, California, USA) for drinking water, surface water and wastewater samples; (2) MO BIO PowerSoil DNA Isolation Kit (MO BIO Laboratories) for mixed faecal samples from poultry pens, soil sample and solid waste samples; and (3) QIAamp DNA Stool Mini Kit (Qiagen, UK) for human stool samples, animal manure and poultry caecal samples. The manufacturer’s protocol supplied with the kit will be followed for extraction of DNA directly from samples.

#### Analysis of samples for detection and quantification of *bla*_NDM-1_ and *bla*_CTX-M-1_ genes by quantitative PCR assay

The amplification and detection of the *bla*_NDM-1_ and *bla*_CTX-M-1_ genes will be carried out in a Bio-Rad CFX96 real-time PCR platform by use of the TaqMan technology. Primers and probe sequences and PCR programs for amplifying *bla*_NDM-1_ and *bla*_CTX-M-1_ genes will be designed inhouse using publicly available sequences in the National Center for Biotechnology Information (NCBI) GenBank. For quantitation of copy number of genes, a standard curve will be generated using known concentrations of selected conserved sequence of both genes obtained from the NCBI GenBank. Using standard gene sequences, gene concentrations will be determined spectrophotometrically using the NanoDrop ND 1000 instrument according to the manufacturer’s instructions (NanoDrop, Wilmington, Delaware). DNA will be 10-fold serially diluted in nuclease-free water (100 ng/mL). Standard curves will be generated using 10^2^–10^7^ copies of standard DNA.

#### Culture of samples for *E. coli* resistance to third-generation cephalosporin or carbapenem

For human and poultry faeces, solid waste and soil samples, 1–5 g of sample will be weighed and dispensed into 9–45 mL of sterile phosphate buffered saline (PBS) in a sterile Falcon tube. After vortexing on high speed for 1 min, the suspension will be allowed to settle at room temperature for 15 min. From the top of the tube, 1 mL of suspension will be extracted and 10-fold serial dilutions will be made using sterile PBS. From each dilution, three aliquots of suspension, each of 0.1 mL, will be taken and inoculated onto one CHROMagar ESBL (CHROMagar, Paris, France), one CHROMagar KPC (*Klebsiella pneumoniae* carbapenemase) (CHROMagar) and one tryptone bile x-glucuronide (TBX) agar plate (Oxoid, UK) following the spread plate technique. All plates will be incubated at 37°C for 18–24 hours. After incubation, the number of typical colonies of *E. coli* on each of the plates (dark pink to reddish colonies on CHROMagar ESBL and KPC plates and blue green colonies on the TBX plates) will be counted. From the ESBL and KPC plates, two typical colonies will be extracted and subcultured on MacConkey agar supplemented with cefotaxime and meropenem, respectively, to obtain pure culture. After overnight incubation of the plates at 37°C, a culture sweep will be taken from each plate and suspended in a cryovial containing tryptic soy broth (TSB) supplemented with 30% glycerol and stored at −80°C until further use.

For water supply samples (tap or tubewell), 2×100 mL of water will be passed separately through two 0.22 µm cellulose membrane filters and the filters will be placed in an upright position on three modified mTEC agar media (BD Difco): one without any supplementation, one supplemented with cefotaxime (1 mg/L) and the other supplemented with meropenem (0.5 mg/L). The plates will be incubated at 37°C for 2 hours followed by 18 hours of incubation at 44°C. The remaining water samples will be kept at 4°C until the test is complete. If the number of colonies is >200 colony-forming units, then appropriate dilutions of the water sample will be made on the following day and the diluted sample will be inoculated on the same media and incubated as above. After incubation, blue colonies, typical of *E. coli*, will be counted. At least two isolated colonies will be extracted from each sample plate and subcultured onto MacConkey agar plate supplemented with the same antibiotic and incubated overnight at 37°C. A culture sweep will be taken from the MacConkey plate and suspended in TSB media with 30% glycerol supplemented with the same antibiotic and kept at −80°C until further use.

#### Confirmation of *E. coli* using API20E

One *E. coli* isolate from each of the samples will be tested using the API20E kit (bioMérieux, France) for biochemical identification. For any sample where the presumptive *E. coli* tests negative according to the API20E test, a second isolate will be tested in order to obtain a confirmed *E. coli* identification.

#### Characterisation of *E. coli* isolates obtained from different sources

All *E. coli* isolates will be tested for different ESBL and CARBase genes including *bla*_CTX-M-1_ and *bla*_NDM-1_. A representative number of isolates from all environmental compartments will be tested for antibiotic susceptibility against different classes of antibiotics following the WHO list of critical antibiotics for human infection. Susceptibility testing will be carried out according to the Clinical and Laboratory Standards Institute guidelines.^32^

*E. coli* isolates will be tested for genetic fingerprinting using random amplified polymorphic DNA (RAPD) analysis following the procedure described earlier.^33^ Isolates will be clustered based on the RAPD banding pattern along with their antibiotic susceptibility patterns. Representative clones from each of the clusters will be analysed further for whole genome sequencing using Illumina MiSeq platform, which allows paired-end, long-read analysis of up to 40 DNA samples per lane. Data will be analysed by an automated custom bioinformatics pipeline.

### Sample size justification

The primary outcome of the study is the prevalence of third-generation cephalosporin and carbapenem antibiotic-resistant *E. coli* isolates and the concentration of ESBL and carbapenem resistance genes in environmental, human and poultry faecal samples.

As a baseline surveillance study to establish the prevalence of resistant bacteria and resistance genes, we have not made an a priori assumption based on the expected difference between high and low exposure groups, or wet and dry season prevalence rates, but this research has been informed by leading research in the field.^4^ The sampling framework is designed to characterise the extent of variability in ABR by selecting 20 farms, 20 markets and 20 rural households in each data collection survey (totalling 40 farms, 40 markets and 40 rural households over two surveys), which will reduce the likelihood of sampling bias.

### Data analysis

- Logistic regression will be carried out to determine whether there are significant differences in the prevalence of colonisation with ESBL-producing or carbapenem-resistant *E. coli* and concentrations of resistance genes in human groups with high and low exposure to environmental contamination and poultry.
- Generalised linear models with robust SEs will be used to identify predictors of colonisation with antibiotic-resistant *E. coli* in humans, such as location (farm, market or rural), occupational exposure, antibiotic use and consumption, and season (wet vs dry) controlling for geographical clustering.
- Analysis of variance (or non-parametric equivalent) will be used to compare the concentrations of resistant bacteria and genes in upstream, wastewater and downstream locations, with spatial mapping of resistant bacteria and gene concentrations.
- A qualitative synthesis will be carried out on the drivers of antibiotic use for poultry by analysis of indepth interviews with farmers, domestic poultry owners and live-bird market workers.
- Logistic regression analysis will be used to compare human exposure-risk behaviours such as handling of poultry, disposal of poultry faeces, contact with poultry carcasses, and solid or liquid waste disposal in each location (markets, farms and rural villages).

## Ethics and dissemination

Data from the project will be hosted by the Environmental Information Data Centre, Centre for Ecology and Hydrology, in line with the data management plan. The results of this study will be published in peer-reviewed journals in the fields of global health, antimicrobial resistance and the natural environment, and presented at national and international conferences.

The research team will follow institutional guidelines on the involvement of human participants and ensure that their rights are protected. We will invite healthy adults (>18 years) to participate in the study, and the study has been designed to be minimally invasive (collection of one stool sample per person) and to take as little time and cause as minimal inconvenience as possible (approximately 20 min for an interviewer-administered questionnaire survey). Observational reports on poultry keeping, selling and slaughtering practices will be carried out in ways that avoid intruding on the daily activities of participants.

Participants will be informed that their involvement is voluntary. The purpose of the study, what would be involved in taking part in the study and the method of collection of faecal samples from participants will be explained in detail on the participant information sheet written in Bangla, and participants will be given opportunities to ask questions or seek clarification. A written informed consent form in Bangla will be obtained from the participants to indicate willingness to take part. Participants will be informed of their right to withdraw from the study. To ensure anonymity, all survey responses and stool samples will be allocated a unique study identification number. No personal identifying details will be kept with either the samples or the survey data.

## Conclusion

This study will provide important baseline data from Bangladesh on the colonisation rates of healthy humans with antibiotic-resistant *E. coli*. Linking human colonisation to occupational and exposure risks from animals and the environment, with simultaneous assessment of resistant bacteria and genes in all environmental compartments, will help to identify and quantify the hot spots for antibiotic-resistant bacteria and antibiotic-resistant genes and the potential risks to human health.

## Supplementary Material

Reviewer comments

Author's manuscript
