# Supplementary material for: Spatial and temporal variation in the community prevalence of antibiotic resistance in Bangladesh: an integrated surveillance study protocol
Source: BMJ Open. 2018 Apr 28;8(4):e023158. doi: 10.1136/bmjopen-2018-023158 (PMC5931287; doi:10.1136/bmjopen-2018-023158)
Supplement: Author's manuscript [file bmjopen-2018-023158.draft_revisions.pdf]

BMJ Open

BMJ Open is committed to open peer review. As part of this commitment we make the peer review history of every article we publish publicly available.

When an article is published we post the peer reviewers' comments and the authors' responses online. We also post the versions of the paper that were used during peer review. These are the versions that the peer review comments apply to.

The versions of the paper that follow are the versions that were submitted during the peer review process. They are not the versions of record or the final published versions. They should not be cited or distributed as the published version of this manuscript.

BMJ Open is an open access journal and the full, final, typeset and author-corrected version of record of the manuscript is available on our site with no access controls, subscription charges or pay-per-view fees (http://bmjopen.bmj.com).

If you have any questions on BMJ Open's open peer review process please email info.bmjopen@bmj.com

BMJ Open

Spatial and temporal variation in the community prevalence of antibiotic resistance in Bangladesh: an integrated surveillance study protocol

Journal:	*BMJ Open*
Manuscript ID	bmjopen-2018-023158.R1
Article Type:	Protocol
Date Submitted by the Author:	09-Apr-2018
Complete List of Authors:	Rousham, Emily; Loughborough University, Centre for Global Health and Human Development, School of Sport, Exercise and Health Sciences<br>Unicomb, Leanne; International Centre for Diarrhoeal Disease Research, Infectious Disease Division<br>Wood, Paul; Loughborough University, Department of Geography<br>Smith, Michael; Loughborough University, Water Engineering and Development Centre, School of Architecture, Building and Civil Engineering<br>Asaduzzaman, Muhammad; International Centre for Diarrhoeal Disease Research, Bangladesh, Laboratory Services and Sciences Division<br>Islam, Mohammad; International Centre for Diarrhoeal Disease Research, Bangladesh, Laboratory Services and Sciences Division
**Primary Subject Heading**:	Global health
Secondary Subject Heading:	Infectious diseases, Epidemiology
Keywords:	PUBLIC HEALTH, Epidemiology < INFECTIOUS DISEASES, Diagnostic microbiology < INFECTIOUS DISEASES

SCHOLARONE™
Manuscripts

Spatial and temporal variation in the community prevalence of antibiotic resistance in Bangladesh: an integrated surveillance study protocol

Authors: Emily Rousham[1], Leanne Unicomb[2], Paul Wood[3], Michael Smith[4], Muhammad Asaduzzaman[5], Mohammad Aminul Islam[5]

[1] Centre for Global Health and Human Development, School of Sport, Exercise and Health Sciences, Loughborough University, UK.

[2] Environmental Intervention Unit, Infectious Diseases Division, International Centre for Diarrhoeal Disease Research, Bangladesh, Dhaka, Bangladesh.

[3] Department of Geography, School of Social, Political and Geographical Sciences, Loughborough University, UK.

[4] Water Engineering and Development Centre, School of Architecture, Building and Civil Engineering, Loughborough University, UK.

[5] Laboratory Sciences and Services Division, International Centre for Diarrhoeal Disease Research, Bangladesh, Dhaka, Bangladesh.

Corresponding author: Dr Emily Rousham, Centre for Global Health and Human Development, School of Sport, Exercise and Health Sciences, Loughborough University, LEICS, LE11 3TU. e.k.rousham@lboro.ac.uk

Joint corresponding author: Mohammad Aminul Islam, Laboratory Sciences and Services Division, icddr, b, GPO Box 128, Dhaka 1000, Bangladesh. maislam@icddrb.org

Word count: 4395

For peer review only

ABSTRACT

Introduction: Increasing antibiotic resistance (ABR) in low and middle-income countries such as Bangladesh presents a major health threat. However, assessing the scale of the health risk is problematic in the absence of reliable data on the community prevalence of antibiotic-resistant bacteria. We describe the protocol for a small-scale integrated surveillance programme that aims to quantify the prevalence of colonisation with antibiotic resistant bacteria and concentrations of antibiotic resistant genes from a 'One Health' perspective. The holistic assessment of antibiotic resistance in humans, animals and within the environment in urban and rural Bangladesh will generate comprehensive data to inform human health risk.

Methods and analysis: The study design focuses on three exposure-relevant sites where there is enhanced potential for transmission of ABR between humans, animals and the environment: i) rural poultry-owning households, ii) commercial poultry farms and iii) urban live-bird markets. The comparison of ABR prevalence in human groups with high and low exposure to farming and poultry will enable us to test the hypothesis that ABR bacteria and genes from the environment and food-producing animals are potential sources of transmission to humans. *Escherichia coli* is used as an ABR indicator organism due to its widespread environmental presence and colonisation in both the human and animal gastrointestinal tract.

Ethics and dissemination: The study has been approved by the Institutional Review Board of the International Centre for Diarrhoeal Disease Research, Bangladesh, (icddr,b) and Loughborough University Ethics Committee. Data for the project will be stored on the open access repository of the Centre for Ecology and Hydrology, Natural Environment Research Council. The results of this study will be published in peer-reviewed journals and presented at national and international conferences.

STRENGTHS AND LIMITATIONS OF THIS STUDY

- We present a study protocol focused on integrated surveillance of ABR in urban and rural Bangladesh using a One Health approach
- Assessment of the human, poultry and environmental prevalence of antibiotic resistant *E. coli* will identify potential hotspots for transmission in Bangladesh

For peer review only - http://bmjopen.bmj.com/site/about/guidelines.xhtml

- Seasonal and spatial variation on the occurrence of antibiotic resistant bacteria in humans, poultry and the wider environment will be assessed
- The two regions included in the study may not be typical of all regions within Bangladesh.

INTRODUCTION

Antibiotic resistance (ABR) has been widely recognised as an emerging threat to human health and a global research priority for capacity development and public health campaigns in clinical and community settings(1). Antimicrobial resistance is seen as a 'One Health' challenge(1). That is, efforts are needed to tackle the problem of ABR in all three domains of humans, animals and in the wider environment in order to achieve optimal human health(2).

The natural environment is acknowledged as an important reservoir of antibiotic resistant bacteria, with anthropogenic activities such as agriculture, antibiotic residues and wastewater disposal potentially enhancing the horizontal transfer of resistant genes(3,4). Genes for antibiotic resistance can survive in the environment, collectively known as the 'resistome', and the exchange of genetic material encoding for antimicrobial resistance can take place through mobile genetic elements such as plasmids via conjugation and other mechanisms in environmental compartments(5). Antimicrobials have been shown also induce the transfer of resistance determinants between microorganisms(6). These processes may be accelerated by the presence of environmental contaminants or pollutants such as heavy metals, antibiotic residues or biocides(7). However, it is not yet known to what extent these environmental sources of antibiotic resistance may be transmitted to humans. There is therefore a pressing need for research to quantify the extent of transmission of clinically relevant ABR bacteria from the environment to humans(8).

Resistance to third-generation cephalosporins is increasing as bacteria acquire the ability to produce extended-spectrum beta-lactamases (ESBLs). The prevalence of colonisation with ESBL-producing *E. coli* is estimated to be around 14% globally, with rates as high as 22% in Asia and sub-Saharan Africa(9). Of greater concern,

For peer review only - http://bmjopen.bmj.com/site/about/guidelines.xhtml

however, is the emergence and spread of carbapenem resistance among clinically relevant bacteria via genes such as New Delhi metallo-β-lactamase-1 (NDM-1) and *mcr*-1(10–12). The detection of these genes in humans, animals and the environment has led to heightened concern regarding the environment as a major transmission route for antimicrobial resistance to humans(10). NDM-1 has been identified in Bangladesh since 2010(13), after the first detection and rapid spread across India and Pakistan from 2008 onwards(14,15). Municipal water supplies in Dhaka are known to carry high levels of sewage-derived bacteria(16). Around 36% of *E. coli* isolates from tap water samples in Dhaka city were reported to be multi-drug resistant and 26% of these were positive for extended spectrum beta-lactamase, posing a serious threat to public health(16). Drinking water contamination is also common in rural areas of Bangladesh; 59% of drinking water samples for children that were stored or directly obtained mostly from tubewells were positive for *E. coli* faecal contamination(17) but were not tested for resistant phenotypes. Prospective sampling indicated that ingestion of contaminated water led to subsequent episodes of diarrhoea among children(17). Approximately 3.5% of gram-negative clinical isolates in Bangladesh are NDM-1-producing(18) and faecal carriage of NDM-1-producing bacteria has been demonstrated among patients with diarrhoea(13,19).

The physical and social environment in Bangladesh favours the rapid spread of ABR with high density populations; lack of clean drinking water and poor sanitation infrastructure. Widespread faecal contamination in water and soil, as well as human hands and food has been demonstrated in microbiological studies in Bangladesh(20). The availability of inexpensive antimicrobials from over-the-counter suppliers leads to inappropriate use of antibiotics(21). The release of antimicrobials into the environment through drug manufacturing and poor waste disposal processes adds further complexities to the pathways for the selection and spread of resistance in the environment(22). In Bangladesh, a high prevalence of $bla_{\text{NDM-1}}$ genes has been found in hospital wastewater compared to community wastewater in the city of Dhaka, highlighting anthropogenic influences on ABR prevalence(23).

As well as the risk factors for ABR associated with water supply, sanitation and infrastructure, the environmental risks of ABR transmission are likely to change with

For peer review only - http://bmjopen.bmj.com/site/about/guidelines.xhtml

seasonal and monsoon rainfall variability, and concomitant changes in human exposure risk to contaminated surface and drinking water(20). Seasonal behaviours may also change transmission dynamics. Ahammad et al.,(24) found high concentrations of *bla*$_{NDM-1}$ in the Yamuna river in Delhi and increased concentration of *bla*$_{NDM-1}$ in rural river water and sediment samples following the mass pilgrimage of visitors from New Delhi to a holy site in the Rishikesh-Haridwar, north India.

We have identified the following gaps in knowledge surrounding the prevalence and transmission of ABR genes and bacteria at the community level: 1. an absence of contemporaneous sampling of ABR in humans, animals and the wider environment in low and middle-income countries such as Bangladesh; 2. a lack of quantitative measures of ABR bacteria (*E. coli*) and resistance genes in potential hot-spots for transmission at an appropriate spatial and temporal scale; 3. an absence of baseline data from rural locations to establish whether ABR prevalence rates are similar to those observed in urban Dhaka; 4. a lack of knowledge regarding the seasonal dynamics of resistant bacteria and gene concentrations in different environmental compartments; and 5. a lack of comparative data of human carriage of resistant genes in groups with high and low exposure to contaminated outdoor environments.

The specific objectives of the research are as follows:

1. To ascertain the prevalence and concentration of antibiotic resistant bacteria (*E. coli*) and resistance genes for ESBLs and carbapenemases (*bla*$_{CTX-M-1}$ and *bla*$_{NDM-1}$) among humans, animal reservoirs (poultry) and different environmental compartments (water, soil and waste) in Bangladesh.
2. To identify the selective drivers of ABR in three exposure-relevant outdoor environments: urban poultry markets; commercial poultry farms, and rural villages.
3. To assess the transfer of resistance within and between microbial communities by comparing ABR carriage rates in humans with high and low exposure to environmental and animal reservoirs of ABR genes.
4. To assess the anthropogenic influences on ABR in the natural environment though ethnographic research among poultry farmers, market sellers and village poultry owners.

For peer review only - http://bmjopen.bmj.com/site/about/guidelines.xhtml

To explore the transmission of ABR between the outdoor environment and human and animal hosts we consider poultry as one of the key drivers of ABR selection through faecal shedding of ABR bacteria. Poultry have high rates of faecal carriage of ABR bacteria; they are most common domestic animal in rural areas of Bangladesh(25) as well as being reared in urban slums in Bangladesh(26). Among poultry owners in rural Bangladesh, 57% reported using over-the-counter antibiotics for their poultry in the last six months; 50% reported sharing their sleeping area with poultry and 61% used poultry faeces as fertilizer(25). The rapid expansion of commercial poultry farming in peri-urban areas carries risks for ABR with high rates of antibiotic use for growth promotion and prophylactic treatment and poor regulation of waste disposal(27,28). Fresh food and poultry markets in Dhaka city are the largest outlets of commercial poultry where birds are slaughtered and processed on site with no regulated solid and liquid waste disposal system(29). Faecal wastes produced within commercial farms and urban markets are mostly disposed into the environment through direct wash out(29).

METHODS AND ANALYSIS

The conceptual framework for the study design and the sampling strategy are shown in Figure 1. The study will generate robust data through a sampling framework targeting three distinct outdoor environments with a high potential for ABR transmission 1) urban live poultry markets in high density residential areas 2) commercial poultry farms in rural sites serving the urban retail market 3) rural villages with traditional animal husbandry and backyard poultry.

E. coli will be investigated as an indicator organism due to its reliability, ease of culturing, affordability, and relevance as a contributor to both diarrheal disease (e.g., diarrheagenic strains) and antibiotic resistance (e.g. multidrug resistant strains). *E. coli* has been listed as a critically important organism for antimicrobial resistance by the WHO(30). The bacterium is a normal resident of the intestinal tract of both human and animals as well as the environment, but pathogenic strains give rise to clinical disease in both hosts(31). Hence, *E. coli* is widely used in studies of the environmental transmission of ABR, particularly for community-acquired resistance using a One Health approach(4).

For peer review only - http://bmjopen.bmj.com/site/about/guidelines.xhtml

The geospatial nature of the transfer of resistance genes will be assessed through geospatial mapping of concentrations of resistant genes and bacteria in upstream localities (water supply), sites close to contamination (source) and downstream (surface waters, soil and solid waste)(see Figure 1) at each location using GIS software (ArcGIS).

Contemporaneous measures will be taken of healthy humans and animal faecal samples. Temporal variability will be assessed by comparison of resistant *E. coli* bacteria and gene concentrations (bla_{NDM-1} and $bla_{CTX-M-1}$) in the dry season compared to wet season in environmental compartments as well as in the human and animal species.

Ethnographic data will be collected regarding customs and practices of market poultry sellers, slaughterers, farm workers and village households that own domestic chickens. Initially, formative observations will be carried out by a trained field team led by an anthropologist followed by in-depth interviews with individuals who work in poultry markets, commercial farms (farmers and suppliers of chicks and feed) or who own backyard/domestic poultry (12-15 interviews of 30-40 minutes duration). The formative observations and in-depth interviews will identify key behaviours relating to human-poultry interactions in each environmental location. Following the formative observations and in-depth interviews, structured observations will be performed to measure behaviours leading to human exposure to poultry and environmental sources of potential contamination. This will yield quantitative data relating to poultry handling, hand washing before and after handling poultry, poultry meat and fresh food preparation, water use and the disposal of solid waste material (poultry faeces and carcasses/feathers). This information, alongside qualitative data from in-depth interviews, will provide contextual data on the human behaviours and practices that may increase environmental contamination and/or increase human exposure to human exposures to contaminated environments and animals.

An interviewer-administered survey of participants will document common practices of domestic (rural) and commercial poultry holders (farmers and market sellers). Domestic and commercial poultry owners and market sellers will be questioned

For peer review only - http://bmjopen.bmj.com/site/about/guidelines.xhtml

about antimicrobial additives in feed; purchase of antimicrobial treatment for animals and the personal purchase and use of antibiotics for themselves.

Selection and recruitment of study participants

Human participants will be recruited to take part in the study on a voluntary basis and will be provided with information about the study. Only those who volunteer and provide their written informed consent will take part in surveys and provide a faecal sample. The sampling within live poultry markets will start from the centre of a market, omitting the first 10 eligible stalls, approaching the next eligible stall and asking the owner to participate. In this way, stallholders with high exposure and their poultry will be recruited. The low exposure group will comprise stallholders without domestic animal produce (e.g. those selling fruit/vegetables) in the same markets. The same geographical sampling strategy will be used for household selection in villages to attain 10 poultry owning and 10 non-poultry owning households. Farms will be selected from a list registered with the Department of Livestock Services and access requested through the Upazila/District Livestock Officer. A low-risk comparison group of non-farm workers will be recruited from the same residential area as the farm workers. Three teams of two fieldworkers will collect samples and data in the wet (monsoon) and dry seasons. Supervision and coordination of fieldwork and laboratory analysis of samples will be carried out by an experienced research investigator. Data collection commenced in March 2017 and is ongoing.

Patient and Public Involvement

As this was a study of healthy adults in community settings, a patient group was not involved in the study design or research protocol. However, the study team consulted local community representatives prior to commencing fieldwork to discuss the research; consulted with the main local hospital, and liaised with local government staff in livestock services to discuss working with the poultry farm workers, as well as obtaining a list of registered commercial farms. This did not result in any direct changes to the protocol but confirmed that the intended methods and approaches were appropriate. Feedback on the research findings will be provided through open meetings in the communities as well as sharing with relevant stakeholders and users of the research as part of the planned dissemination activities.

For peer review only - http://bmjopen.bmj.com/site/about/guidelines.xhtml

Sample collection for laboratory analysis

Poultry caecal samples

Live chickens will be collected from rural households, poultry farms and live bird markets and slaughtered. After defeathering, the whole carcass will be placed in a sterile zipper bag. After transportation to the laboratory under a cool chain (4^oC to 8^oC) caecal samples will be collected aseptically from the dissected whole carcass.

Poultry faeces samples

Poultry litter samples will be collected from three different areas of the bird cage/container; floor (in cases where birds are kept in a confined area and not in a cage) or grounds of a compound for free-ranging animals and pooled in a sterile plastic bag and mixed thoroughly.

Solid waste samples

Solid waste samples will be collected from the municipal waste disposal sites near the markets. Samples will be collected from three different locations surrounding the waste collection area. A sterile spatula or spoon will be used to collect solid waste (dark soil) to a depth of 2.5-5.0 cm, pooled in a sterile plastic bag and mixed thoroughly.

Soil samples

Soil samples will be collected from within rural compounds/households. For households with internal floors made of mud-compacted soil, a sample will be collected from the main room of the house where most people usually sleep. The area should be dry and free of visible faeces, food, or waste. In houses with concrete floors we will choose an area in the yard where there is frequent movement of people (e.g. near the kitchen or in front of the main household door). Using an autoclaved spoon, a maximum depth of about 1.5 cm below the soil surface will be excavated covering an area of ~ 30 cm^2. Around 150 g of soil will be collected in a labelled sterile sample bag. Using the end of the spoon, a small scoop (about 0.25 g) of soil from the same area will be placed into a cryovial with LifeGuard solution.

Waste water samples

For peer review only - http://bmjopen.bmj.com/site/about/guidelines.xhtml

Waste water samples will be collected from three separate locations of the run-off drain adjacent to the farm, household or market and pooled together in a sterile plastic bottle (Nalgene, Rochester, NY). Approximately 300 ml of water will be collected from each point by submerging a sterile container into the drain and transferred to a 1000 ml sterile plastic bottle.

Surface water samples

Water samples from rivers, ponds or other surface water downstream of peri-urban and rural areas will be collected using a sterile plastic bottle. A bottle will be filled by plunging downwards about 30 cm below the water surface and resealed tightly to prevent leakage. Particular care will be taken to avoid bacterial contamination inside the bottle or cap from sources other than the water. Sample bottles will be transported to the laboratory within 8h of collection.

Supply water samples

To collect water sample from tubewells, the hand pump will be operated continuously for 5 minutes. The mouth of the pump will then be heated by means of a gas torch, followed by pumping several litres of water to waste. A sample of water will be collected aseptically by allowing the water from the pump to flow directly into a sterile bottle. After collection, the cap of the bottle will be replaced carefully.

To collect samples from taps, the outside nozzle of the tap will be cleaned carefully. The tap will then be turned on full and the water allowed to run to waste for 1 minute. The outside of the tap will be sterilized using the flame of a gas torch and allowed to cool by running the water to waste for a few seconds. The sterile sample bottle will be filled from a gentle flow of water and the cap placed on the bottle. Sample bottles will be transported to the laboratory within 8h of collection.

Human stool samples

Each study participant will be provided with a stool sample container and the procedure for the collection of samples will be explained. Field staff will pick up the sample container within 2 hours of collection.

For peer review only - http://bmjopen.bmj.com/site/about/guidelines.xhtml

For all solid and liquid samples collected in the field, sample bags, sample bottles and vials will be immediately placed in a cool box ($4^{o}C$ to $8^{o}C$) and transported to the laboratory for analysis.

Laboratory processing and analysis of human, animal and environmental samples

DNA will be extracted directly from samples using the following kits: 1. MO Bio Power Water DNA isolation Kit (MO BIO Laboratories, Inc., CA, USA) for drinking water, surface water, waste water samples; 2. MO Bio Power Soil DNA isolation Kit (MO BIO Laboratories, Inc., CA, USA) for mixed faecal samples from poultry pens, soil sample, and solid waste samples and 3. QIAamp DNA Stool Mini Kit (Qiagen, UK) for human stool samples, animal manure and poultry caecal samples. The manufacturer's protocol supplied with the kit will be followed for extraction of DNA directly from samples.

Analysis of samples for detection and quantification of bla_{NDM-1} and $bla_{CTX-M-1}$ genes by qPCR assay

The amplification and detection of the bla_{NDM-1} and $bla_{CTX-M-1}$ genes will be carried out in a Bio-Rad CFX 96 real-time PCR platform by use of the TaqMan technology. Primers and probe sequences and PCR programs for amplifying bla_{NDM-1} and $bla_{CTX-M-1}$ genes will be designed in house using publicly available sequences in the NCBI genbank. For quantitation of copy number of genes, a standard curve will be generated using known concentrations of selected conserved sequence of both genes obtained from the NCBI genbank. Using standard gene sequences, gene concentrations will be determined spectrophotometrically using the Nanodrop ND 1000 instrument according to the manufacturer's instructions (Nanodrop, Wilmington, DE). DNA will be 10-fold serially diluted in nuclease-free water (100ng/microliter). Standard curves will be generated using 10^2 to 10^7 copies of standard DNA.

Culture of samples for *E. coli* resistance to third generation cephalosporin or carbapenem

For human and poultry faeces, solid waste and soil samples, 1–5 g of sample will be weighed and dispensed into 9-45 ml of sterile PBS in a sterile Falcon tube. After vortexing on high speed for one minute, the suspension will be allowed to settle at

For peer review only - http://bmjopen.bmj.com/site/about/guidelines.xhtml

room temperature for 15 minutes. From the top of the tube, 1 ml of suspension will be extracted and 10-fold serial dilutions will be made using sterile PBS. From each dilution, three aliquots of suspension, each of 0.1 ml, will be taken and inoculated onto one CHROMagar ESBL (CHROMagar, Paris, France); one CHROMagar KPC (CHROMagar, Paris, France), and one TBX agar plate (Oxoid Ltd., UK) following spread plate technique. All plates will be incubated at 37°C for 18-24h. After incubation, the number of typical colonies of *E. coli* on each of the plates (dark pink to reddish colonies on ESBL and KPC plates and blue green colonies on the TBX plates) will be counted. From the ESBL and KPC plates, two typical colonies will be extracted and sub-cultured on MacConkey agar supplemented with cefotaxime and meropenem, respectively to obtain pure culture. After overnight incubation of the plates at 37°C, a culture sweep will be taken from each plate and suspended in a cryovial containing TSB supplemented with 30% glycerol and stored at -80°C until further use.

For water supply samples (tap or tubewell), 2 x 100 ml of water will be passed separately through two 0.22 µm cellulose membrane filters and the filters will be placed in an upright position on three modified mTEC agar media (BD Difco®); one without any supplementation, one supplemented with cefotaxime (1 mg/L) and the other supplemented with meropenem (0·5 mg/L). The plates will be incubated at 37°C for 2h followed by 18h of incubation at 44°C. The remaining water samples will be kept at 4°C until the test is complete. If the number of colonies is >200 CFU then appropriate dilutions of the water sample will be made on the following day and the diluted sample will be inoculated on the same media and incubated as above. After incubation, blue colonies, typical of *E. coli,* will be counted. At least two isolated colonies will be extracted from each sample plate and subcultured onto MacConkey agar plate supplemented with the same antibiotic and incubated overnight at 37°C. A culture sweep will be taken from the MacConkey plate and suspended in TSB media with 30% glycerol supplemented with the same antibiotic and kept at -80°C until further use.

Confirmation of *E. coli* using API 20E

One *E. coli* isolate from each of the samples will be tested using the API20E kit (BioMérieux, France) for biochemical identification. For any sample where the

For peer review only - http://bmjopen.bmj.com/site/about/guidelines.xhtml

presumptive *E. coli* tests negative according to the API20E test, a second isolate will be tested in order to obtain a confirmed *E. coli* identification.

Characterisation of *E. coli* isolates obtained from different sources

All *E. coli* isolates will be tested for different ESBL and CARBase genes including $bla_{\text{CTX-M-1}}$ and $bla_{\text{NDM-1}}$. A representative number of isolates from all environmental compartments will be tested for antibiotic susceptibility against different classes of antibiotics following the WHO list of critical antibiotics for human infection. Susceptibility testing will be carried out according to CLSI guidelines (32).

E. coli isolates will be tested for genetic fingerprinting using random amplified polymorphic DNA (RAPD) analysis following the procedure described earlier (33). Isolates will be clustered based on the RAPD banding pattern along with their antibiotic susceptibility patterns. Representative clones from each of the clusters will be analysed further for whole genome sequencing using Illumina MiSeq platform, which allows paired-end, long-read analysis of up to 40 DNA samples per lane. Data will be analysed by an automated custom bioinformatics pipeline.

Sample size justification

The primary outcome of the study is the prevalence of third generation cephalosporin and carbapenem antibiotic resistant *E. coli* isolates and the concentration of ESBL and carbapenem resistance genes in environmental, human and poultry faecal samples.

As a baseline surveillance study to establish the prevalence of resistant bacteria and resistance genes we have not made an *a priori* assumption based on the expected difference between high and low exposure groups, or wet and dry season prevalence rates, but this research has been informed by leading research in the field(4). The sampling framework is designed to characterise the extent of variability in ABR by selecting 20 farms, 20 markets and 10 villages in each data collection survey (totalling 40 farms, 40 markets and 20 villages over two surveys), which will reduce the likelihood of sampling bias.

Data Analysis

For peer review only - http://bmjopen.bmj.com/site/about/guidelines.xhtml

- Logistic regression will be carried out to determine whether there are significant differences in the prevalence of colonisation with ESBL-producing or carbapenem resistant *E. coli* and concentrations of resistance genes in human groups with high and low exposure to environmental contamination and poultry.
- Generalised linear models with robust standard errors will be used to identify predictors of colonisation with antibiotic resistant *E. coli* in humans such as location (farm, market or rural), occupational exposure, antibiotic use and consumption, and season (wet versus dry) controlling for geographical clustering.
- Analysis of variance (or non-parametric equivalent) will be used to compare the concentrations of resistant bacteria and genes in upstream, wastewater and downstream locations, with spatial mapping of resistant bacteria and gene concentrations.
- A qualitative synthesis will be carried out on the drivers of antibiotic use for poultry by analysis of in-depth interviews with farmers, domestic poultry owners and live bird market workers.
- Logistic regression analysis will be used to compare human exposure-risk behaviours such as handling of poultry, disposal of poultry faeces, contact with poultry carcasses and solid or liquid waste disposal in each location (markets, farms and rural villages).

ETHICS AND DISSEMINATION

This research protocol was submitted to the Institutional Review Board of icddr,b, Bangladesh, where it received clearance from the Research Review Committee and Ethical Review Committee (protocol number PR-16071). Approval from the Human Participants Ethical sub-committee was also obtained from Loughborough University (R17-P037).

Data from the project will be hosted by the Environmental Information Data Centre, Centre for Ecology and Hydrology in line with the data management plan. The results of this study will be published in peer-reviewed journals in the fields of global

For peer review only - http://bmjopen.bmj.com/site/about/guidelines.xhtml

health; antimicrobial resistance and the natural environment and presented at national and international conferences.

The research team will follow institutional guidelines on the involvement of human participants and ensure that their rights are protected. We will invite healthy adults (>18 years) to participate in the study, and the study has been designed to be minimally invasive (collection of one stool sample per person) and to take as little time and cause as minimal inconvenience as possible (approximately 20 minutes for an interviewer-administered questionnaire survey). Observational reports on poultry-keeping, selling and slaughtering practices will be carried out in ways that avoid intruding on the daily activities of participants.

Participants will be informed that their involvement is voluntary. The purpose of the study, what would be involved in taking part in the study, and the method of collection of faecal samples from participants will be explained in detail in the participant information sheet written in Bangla, and participants will be given opportunities to ask questions or seek clarification. Information about the study will be provided in the form of a participant information letter in Bangla (to be read verbally and given in writing to participants). A written informed consent form in Bangla will be obtained from the participants to indicate willingness to take part. Participants will be informed of their right to withdraw from the study. To ensure anonymity, all survey responses and stool samples will be allocated a unique study identification number. No personal identifying details will be kept with either the samples or the survey data.

For peer review only

CONCLUSION

This study will provide important baseline data from Bangladesh on the colonisation rates of healthy humans with antibiotic resistant *E. coli*. Linking human colonisation to occupational and exposure risks from animals and the environment, with simultaneous assessment of resistant bacteria and genes in all environmental compartments, will help to identify and quantify the hotspots for antibiotic-resistant bacteria and antibiotic-resistant genes and the potential risks to human health.

For peer review only - http://bmjopen.bmj.com/site/about/guidelines.xhtml

Authors' contributions: EKR, MAI, LU, PW and MS all contributed to the design and development of the study protocol. MA contributed to the field methods, site selection and laboratory protocols. All authors contributed to writing the final protocol and manuscript.

Funding statement: This work was supported by the Antimicrobial Resistance Cross Council Initiative supported by the seven research councils in partnership with the Department of Health and Department for Environment Food & Rural Affairs (NERC/BBSRC/MRC grant number: NE/N019555/1).

Competing interests statement: The authors declare no competing interests.

References

1. O 'Neill J. Tackling Drug-Resistant Infections Globally: Final Report and Recommendations the Review on Antimicrobial Resistance. London. 2016; Available at: https://amr-review.org/sites/default/files/160518_Final%20paper_with%20cover.pdf. Accessed 20 Mar 2018
2. King LJ, Anderson LR, Blackmore CG, et al. Executive summary of the AVMA One Health Initiative Task Force report. *J Am Vet Med Assoc*. 2008;233:259–61. doi.10.2460/javma.233.2.259
3. Amos GCA, Hawkey PM, Gaze WH, et al. Waste water effluent contributes to the dissemination of CTX-M-15 in the natural environment. *J Antimicrob Chemother*. 2014;69:1785–91. doi.10.1093/jac/dku079
4. Pehrsson EC, Tsukayama P, Patel S, et al. Interconnected microbiomes and resistomes in low-income human habitats. *Nature*. 2016 12;533:212–6. doi.org/10.1038/nature17672
5. Wellington EMH, Boxall ABA, Cross P, et al. The role of the natural environment in the emergence of antibiotic resistance in Gram-negative bacteria. *Lancet Infect Dis*. 2013;13:155–65. doi.org/10.1016/S1473-3099(12)70317-1
6. Holmes AH, Moore LSP, Sundsfjord A, et al. Understanding the mechanisms and drivers of antimicrobial resistance. *Lancet*. 2016;387:176–87. doi.10.1016/S0140-6736(15)00473-0

For peer review only - http://bmjopen.bmj.com/site/about/guidelines.xhtml

7. Pruden A, Larsson JDG, Amézquita A, et al. Management options for reducing the release of antibiotics and antibiotic resistance genes to the environment. *Environ Health Perspect*. 2013;121:878–85. doi.10.1016/S0140-6736(15)00473-0

8. Huijbers PMC, Blaak H, De Jong MCM, et al. Role of the Environment in the Transmission of Antimicrobial Resistance to Humans: A Review. *Environ Sci Technol*. 2015;49:11993–2004. doi.10.1021/acs.est.5b02566

9. Karanika S, Karantanos T, Arvanitis M, et al. Fecal colonization with extended-spectrum beta-lactamase-producing Enterobacteriaceae and risk factors among healthy Individuals: A systematic review and metaanalysis. *Clin Infect Dis*. 2016;63:310–8. doi.10.1093/cid/ciw283

10. Kumarasamy KK, Toleman MA, Walsh TR, et al. Emergence of a new antibiotic resistance mechanism in India, Pakistan, and the UK: A molecular, biological, and epidemiological study. *Lancet Infect Dis*. 2010;10:597–602. doi.10.1016/S1473-3099(10)70143-2

11. Liu YY, Wang Y, Walsh TR, et al. Emergence of plasmid-mediated colistin resistance mechanism MCR-1 in animals and human beings in China: A microbiological and molecular biological study. *Lancet Infect Dis.* 2016;16:161–8. doi.10.1016/S1473-3099(15)00424-7

12. Jeannot K, Bolard A, Plésiat P. Resistance to polymyxins in Gram-negative organisms. *Int J Antimicrob Agents*. 2017;49:526–35. doi.10.1016/j.ijantimicag.2016.11.029

13. Islam MA, Huq M, Nabi A, et al. Occurrence and characterization of multi-drug resistant New Delhi metallo-β-lactamase-1-producing bacteria isolated between 2003 and 2010 in Bangladesh. *J Med Microbiol*. 2013;62(PART1):62–8. doi.10.1099/jmm.0.048066-0

14. Yong D, Toleman MA, Giske CG, et al. Characterization of a new metallo-β-lactamase gene, blaNDM-1, and a novel erythromycin esterase gene carried on a unique genetic structure in Klebsiella pneumoniae sequence type 14 from India. *Antimicrob Agents Chemother*. 2009;53:5046–54. doi.10.1128/AAC.00774-09

15. Walsh TR, Weeks J, Livermore DM, et al. Dissemination of NDM-1 positive bacteria in the New Delhi environment and its implications for human health: An environmental point prevalence study. *Lancet Infect Dis*. 2011;11:355–62.

For peer review only - http://bmjopen.bmj.com/site/about/guidelines.xhtml

doi.10.1016/S1473-3099(11)70059-7

16. Talukdar PK, Rahman M, Rahman M, et al. Antimicrobial resistance, virulence factors and genetic diversity of *Escherichia coli* isolates from household water supply in Dhaka, Bangladesh. *PLoS One*. 2013;8. doi.10.1371/journal.pone.0061090

17. Luby SP, Halder AK, Huda TM, et al. Microbiological contamination of drinking water associated with subsequent child diarrhea. *Am J Trop Med Hyg*. 2015;93(5):904–11. doi.10.4269/ajtmh.15-0274

18. Islam MA, Talukdar PK, Hoque A, et al. Emergence of multidrug-resistant NDM-1-producing Gram-negative bacteria in Bangladesh. *Eur J Clin Microbiol Infect Dis*. 2012;31:2593–600. doi.10.1007/s10096-012-1601-2

19. Islam MA, Nabi A, Rahman M, et al. Prevalence of faecal carriage of NDM-1-producing bacteria among patients with diarrhoea in Bangladesh. *J. Med. Microbiol*. 2014;63:620–2. doi:10.1099/jmm.0.064527-0

20. Ercumen A, Pickering AJ, Kwong LH, et al. Animal feces contribute to domestic fecal contamination: evidence from *E. coli* measured in water, hands, food, flies, and soil in Bangladesh. *Environ Sci Techno*l. 2017;51:8725–34. doi. 10.1021/acs.est.7b01710

21. Bloom G, Wilkinson A, Tomson G, et al. AMR Addressing Resistance To Antibiotics In Pluralistic Health Systems. The STEPS Centre. Brighton; 2015.

22. Lübbert C, Baars C, Dayakar A, et al. Environmental pollution with antimicrobial agents from bulk drug manufacturing industries in Hyderabad, South India, is associated with dissemination of extended-spectrum beta-lactamase and carbapenemase-producing pathogens. *Infection*. 2017;45:479-491. doi.10.1007/s15010-017-1007-2

23. Islam MA, Islam M, Hasan R, et al. Environmental spread of NDM-1-producing multi-drug resistant bacteria in Dhaka, Bangladesh. *Appl Environ Microbiol*. 2017;AEM.00793-17. doi.10.1128/AEM.00793-17

24. Ahammad ZS, Sreekrishnan TR, Hands CL, et al. Increased waterborne bla NDM-1 resistance gene abundances associated with seasonal human pilgrimages to the Upper Ganges River. *Environ Sci Technol* 2014;48:3014–20. doi.10.1021/es405348h

25. Roess AA, Winch PJ, Akhter A, et al. Household animal and human medicine use and animal husbandry practices in rural Bangladesh: risk factors for

For peer review only - http://bmjopen.bmj.com/site/about/guidelines.xhtml

emerging zoonotic disease and antibiotic resistance. *Zoonoses Public Health*. 2015;62:569–78. doi.10.1111/zph.12186

26. Harris AR, Pickering AJ, Harris M, et al. Ruminants contribute fecal contamination to the urban household environment in Dhaka, Bangladesh. *Environ Sci Technol.* 2016;50:4642–9. doi.10.1021/acs.est.5b06282
27. Braykov NP, Eisenberg JNS, Grossman M, et al. Antibiotic resistance in animal and environmental samples associated with small-scale poultry farming in northwestern Ecuador. *mSphere*. 2016;1:e00021-15. doi.10.1128/mSphere.00021-15
28. Brower CH, Mandal S, Hayer S, et al. The prevalence of extended-spectrum beta-lactamase-producing multidrug-resistant *Escherichia coli* in poultry chickens and variation according to farming practices in Punjab, India. *Environ Health Perspect*. 2017;125:1–10. doi.10.1289/EHP292
29. UNICEF and ICDDRB. Evaluation of Avian Influenza Communication for Development Initiative- Improving Biosecurity in Live Bird Markets. 2015. Dhaka: icddr,b. Pp160. https://www.unicef.org/cbsc/files/Avian_Campaign_Eval-Bangladesh-2014.pdf Accessed 20 Mar 2018
30. Knols BG, Smallegange RC, Tacconelli E, et al. Global Priority List of Antibiotic-Resistant Bacteria to Guide Research, Discovery, and Development of New Antibiotics. World Health Organization. 2017. http://www.who.int/medicines/publications/global-priority-list-antibiotic-resistant-bacteria/en/. Accessed 20 Mar 2018
31. Aarestrup FM, Wegener HC, Collignon P. Resistance in bacteria of the food chain: Epidemiology and control strategies. *Expert Rev Anti Infect Ther*. 2008;6:733–50. doi:10.1586/14787210.6.5.733
32. The Clinical and Laboratory Standards Institute. Performance Standards for Antimicrobial Susceptibility Testing CLSI supplement M100S. Clinical and Laboratory Standards Institute, Wayne, PA. 2016. 256 p.
33. Chansiripornchai N, Ramasoota P, Sasipreeyajan J, et al. Differentiation of avian pathogenic Escherichia coli (APEC) strains by random amplified polymorphic DNA (RAPD) analysis. *Vet Microbiol*. 2001;80:75–83. doi.10.1016/S0378-1135(00)00380-1

For peer review only - http://bmjopen.bmj.com/site/about/guidelines.xhtml

For peer review only

For peer review only - http://bmjopen.bmj.com/site/about/guidelines.xhtml

Figure Legends

Figure 1: Sampling framework showing the three study sites in relation to the research objectives.

Footnote to Figure 1: In each site, one sampling round will be conducted in the dry season (n=20 markets; n=20 farms; n=20 households) and another sampling round in the wet season (an additional n=20 markets; n=20 farms; n=20 households).

For peer review only

For peer review only - http://bmjopen.bmj.com/site/about/guidelines.xhtml

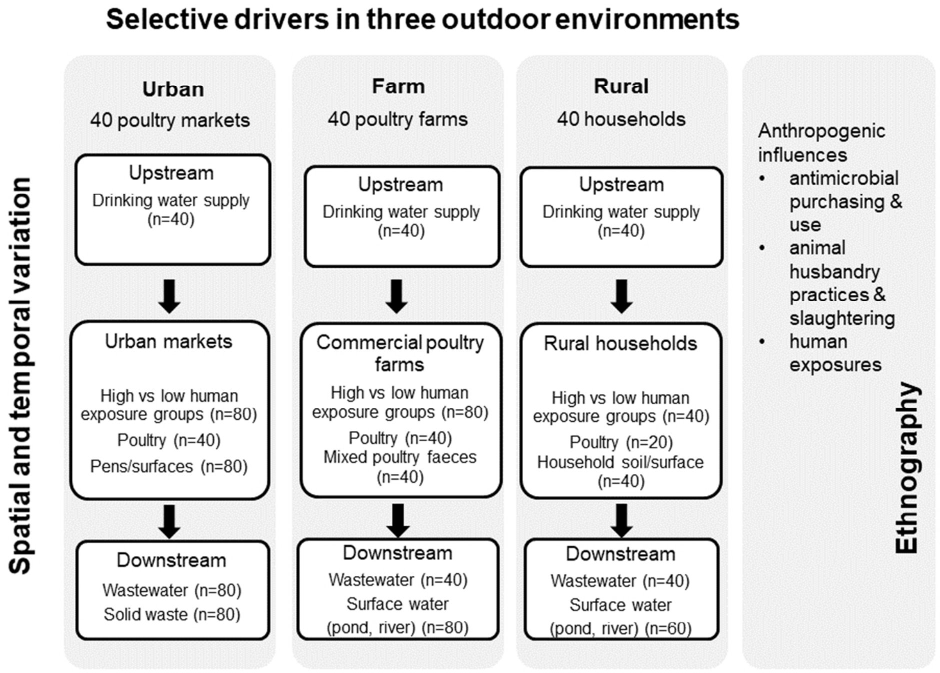

Figure 1: Sampling framework showing the three study sites in relation to the research objectives.
Footnote to Figure1: In each site, one sampling round will be conducted in the dry season (n=20 markets; n=20 farms; n=20 households) and another sampling round in the wet season (an additional n=20 markets; n=20 farms; n=20 households).
