# Supplementary material for: Spatial and temporal variation in the community prevalence of antibiotic resistance in Bangladesh: an integrated surveillance study protocol
Source: BMJ Open. 2018 Apr 28;8(4):e023158. doi: 10.1136/bmjopen-2018-023158 (PMC5931287; doi:10.1136/bmjopen-2018-023158)
Supplement: Reviewer comments [file bmjopen-2018-023158.reviewer_comments.pdf]

PEER REVIEW HISTORY

BMJ Open publishes all reviews undertaken for accepted manuscripts. Reviewers are asked to complete a checklist review form (**http://bmjopen.bmj.com/site/about/resources/checklist.pdf**) and are provided with free text boxes to elaborate on their assessment. These free text comments are reproduced below.

ARTICLE DETAILS

**TITLE (PROVISIONAL)**	Spatial and temporal variation in the community prevalence of antibiotic resistance in Bangladesh: an integrated surveillance study protocol
**AUTHORS**	Rousham, Emily; Unicomb, Leanne; Wood, Paul; Smith, Michael; Asaduzzaman, Muhammad; Islam, Mohammad

This article was not externally reviewed at BMJ Open. Protocols that have been independently assessed prior to submission to BMJ Open are usually fast-tracked to publication on the grounds that further substantial changes will not be possible. This independent assessment will usually be external, independent review for both a substantial grant award from a non-commercial or government funder and ethics approval.
